# A Rare Cause of Optic Neuropathy

**DOI:** 10.7759/cureus.6906

**Published:** 2020-02-07

**Authors:** Isabel O Cruz, Sara Alves Pereira, Bruna Vieira, Inês Chora, Paulo Coelho

**Affiliations:** 1 Internal Medicine, Hospital Pedro Hispano, Porto, PRT; 2 Ophthalmology, Hospital Pedro Hispano, Matosinhos, PRT; 3 Ophtalmology, Hospital Pedro Hispano, Matosinhos, PRT; 4 Internal Medicine, Hospital Pedro Hispano, Matosinhos, PRT; 5 Neurology, Hospital Pedro Hispano, Matosinhos, PRT

**Keywords:** lyme disease, optic neuritis, meningitis, borrelia

## Abstract

Lyme disease is a multisystem infection caused by Borrelia burgdorferi that mainly affects the joints, the heart, and the nervous system. Neurological complications usually manifest in untreated patients and present as meningitis, cranial neuropathies, and radiculoneuritis.

The authors present the case of a 48-year-old male who developed loss of vision in the right eye over a period of two months. On physical examination a relative afferent pupillary defect of the right eye was noted. Visual evoked potential test revealed delayed P100 latency bilaterally, confirming a bilateral optic neuropathy. The analysis of the cerebrospinal fluid (CSF) showed a lymphocytic meningitis. After an extensive work-up, a diagnosis of Lyme neuroborreliosis with meningitis and optic neuritis was made. The patient was treated with antibiotics and showed gradual improvement. The follow-up brain MRI revealed a mild T2 hyperintensity on the right optic nerve with gliosis, sequelae of the inflammatory process.

Lyme disease should always be considered in patients from endemic areas with nonspecific symptoms. The diagnosis of neuroborreliosis is challenging, but prompt identification and treatment can prevent the development of complications and sequelae.

## Introduction

Lyme disease is a zoonotic disease caused by *Borrelia burgdorferi* sensu lato complex, a group of flagellated spirochete species, transmitted to humans through bites from infected ticks of the Ixodes species [1]. It is the most common arthropod-borne disease in the northern hemisphere [2]. The species known to cause human disease are *B. afzelii*, *B. burgdorferi* sensu strict, and *B. garinii* [3].

The diagnosis of Lyme disease is challenging because of its various presentations. It is characterized by three stages: 1) early localized disease, which occurs in the first month after an infected tick bite and whose main feature is erythema migrans, seen in 50%-70% of patients [4], and flu-like symptoms; 2) early disseminated disease, one to four months after the bite, due to hematogeneous dissemination of the bacteria; and 3) late disease, four months to years later. The clinical picture varies with the organ affected, the most common being the heart, the joints, and the nervous system.

It is estimated that 15% of people with untreated Lyme disease will develop neurological manifestations [5]. The early stage of neuroborreliosis typically presents in the summer and early fall and includes lymphocytic meningitis, cranial neuropathies (mainly facial nerve palsy), and radiculoneuritis [3]. The late stages are characterized by peripheral neuropathies, chronic encephalomyelitis, and mild encephalopathy [3]. The mechanisms of nervous system involvement are unknown, but probably include neurotoxic mediators, immunological responses, and vasculitis [3].

Optic neuritis is an inflammation of the optic nerve resulting in blurred vision, eye pain, a central visual field defect, dyschromatopsia, and a relative afferent pupillary defect. It is common in autoimmune, inflammatory, and infectious diseases of the central nervous system (CNS) but its association with Lyme disease is poorly understood [6]. Few cases of optic neuritis due to borreliosis have been published in the literature. Although rare, it should be included in the differential diagnosis of optic neuritis in endemic areas.

## Case presentation

A 48-year-old Portuguese man living in Switzerland, without relevant past personal or family history, presented to the ED during late summer, complaining of gradual vision loss in the right eye. The patient reported a two-month history of blurred vision, which initially involved the inferior field of vision, but later progressed to involve the entire visual field. He also reported mild right-sided headaches and photopsia. He denied any other symptoms. He was a bricklayer and his most recent work consisted of a railway construction in a forested area near Lake Geneva. No other epidemiological risk factors were identified.

**Figure 1:**
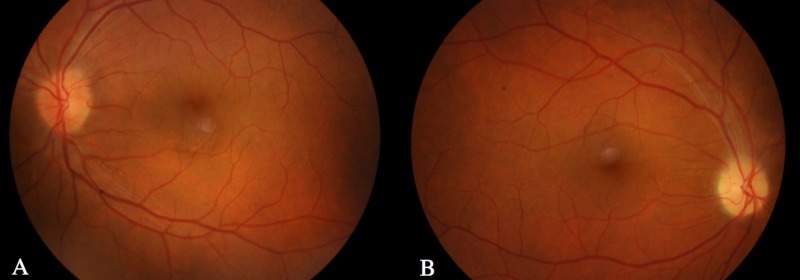
Retinography. A – Left eye with hyperemia and diffuse edema of the disc. B – Right eye with pale optic disc and edema of the inferior quadrant. Note the absence of hemorrhages and exudates. Both eyes exhibited retinal vessel tortuosity.

Physical examination, including a complete neurological exam, was only remarkable for a relative afferent pupillary defect of the right eye. Visual acuity was 10/10 in both eyes. The anterior segment was normal on ultrasound biomicroscopy. Dilated fundus examination showed a pale optic disc and edema of the inferior quadrant of the right eye and hyperemia and diffuse edema on the left (Figure 1).

**Figure 2:**
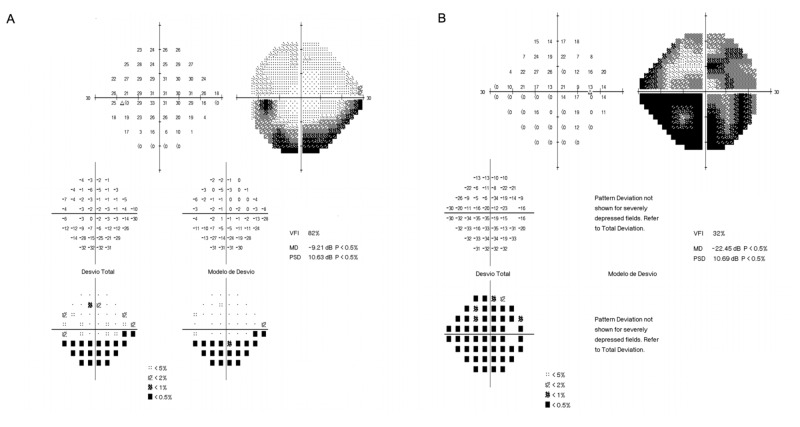
Visual field test. A - Left eye. B - Right eye.

Retinal examination was normal. Admission laboratory results were within reference limits, except for C reactive protein, which was slightly elevated at 19.7 mg/L (normal range: <5.0 mg/L). Cerebral CT scan with contrast was normal. Visual field testing revealed a generalized deficit on the right eye and a left inferior scotoma (Figure 2).

Fluorescein angiography and optic disc optical coherence tomography (OCT) confirmed the disc edema and the macular OCT was normal. Visual evoked potential test revealed delayed P100 latency bilaterally. The patient was hospitalized to study the etiology of his optic neuropathy.

The patient had normal results for other tests, including lipid profile, thyroid function, sedimentation rate, and angiotensin conversion enzyme. Some infections were excluded (Venereal Disease Research Laboratory (VDRL) test, HIV 1 and 2 antibodies, Hepatitis C antibody, Hepatitis B surface antigen and antibody, Wright reaction were negative). Immunological blood tests such as antinuclear and anti-neutrophil cytoplasmic antibodies, anti-dsDNA antibodies, extractable nuclear antigen antibodies panel, C3 and C4 complement, rheumatoid factor, cardiolipin antibodies, beta-2 glycoprotein antibodies, lupus anticoagulant, anti-aquaporin-4, and anti-MOG (myelin oligodendrocyte glycoprotein) antibodies were normal. Lumbar puncture revealed normal opening pressure and cerebrospinal fluid (CSF) analysis showed pleocytosis (177 cells/mL) with predominance of mononuclear cells and elevated protein levels (185.4 mg/dL, normal range of 15.0-45.0 mg/dL). Bacterial cultures were negative and Mycobacterium infection was excluded by polymerase chain reaction (PCR), Ziehl-Neelsen stain, and cultures. Immunoglobulin G (IgG) oligoclonal bands were detected in the CSF but not in serum, pointing to intrathecal synthesis of IgG and CNS inflammation. Brain and orbit magnetic resonance imaging was unremarkable.

Serum immunoassay for borrelia by enzyme-linked immunosorbent assay (ELISA) showed elevated levels of IgG and IgM antibodies (>200 and 195, respectively, with a positive cut-off of 22). The diagnosis was further confirmed by Western Blot and four bands were positive (p41 and VIsE for IgG and p41 and OspC for IgM). PCR for borrelia in CSF was negative, but this test has a low sensitivity.

The patient was diagnosed with meningitis and bilateral optic neuritis secondary to Lyme disease. In 2005, Sibony et al. proposed criteria for strong evidence of optic neuritis associated with active Lyme disease (Table 1), which our patient fulfilled [7].

**Table 1:** Sibony’s criteria for strong evidence of optic neuritis associated with active Lyme disease diagnosis. * Criteria the reported case fulfilled. VDRL – Venereal Disease Research Laboratory, RPR – Rapid plasma reagin, ELISA – Enzyme-linked immunosorbent assay, IFA – Indirect fluorescent antibody, PCR – Polymerase chain reaction, WB – Western Blot, CSF – Cerebrospinal fluid.

Strong evidence requires the following core elements:
Optic neuritis *
Endemic exposure *
Negative VDRL or RPR test *
Exclusion of multiple sclerosis *
Positive Lyme titer (ELISA or IFA) *
Plus one of the following:
Encephalitis / Meningitis with CSF pleocytosis, intrathecal antibody production or CSF PCR or WB positive for B. burgdorferi *
Recent Lyme disease signs (facial nerve palsy, arthritis, or radiculoneuritis) with positive serum ELISA or WB
Recent diagnosis of erythema migrans by a physician, usually with associated flu-like symptoms

He reported no joint pain and had no signs of arthritis; heart involvement was excluded by normal electrocardiogram and echocardiogram. Treatment with a 28-day course of intravenous ceftriaxone 2 g twice daily led to symptomatic improvement.

On further questioning, the patient did not recall any tick bite wound but he did remember finding ticks on his clothes.

After discharge, the patient went back to Switzerland and therefore was lost to follow up.

## Discussion

This is a rare case of CNS involvement in early Lyme disease, presenting with meningitis and bilateral optic neuritis.

The diagnosis of Lyme disease requires a high level of suspicion and only a minority of patients recall tick bites or cutaneous lesions. The most important preventive measure remains educating the population about Lyme Borreliosis and how to avoid tick bites. A previous infection does not provide protective immunity and reinfections may occur [8].

The incidence of Lyme disease in some areas of Switzerland is among the highest in Europe, according to the International Association for Medical Assistance to Travellers (IAMAT), making it an endemic zone. The Centers for Disease Control (CDC) recommends that serologic testing for Lyme disease should only be considered positive when both ELISA and Western Blot analyses are positive [9]. If CNS involvement is suspected, CSF can be analyzed for intrathecal antibody production or detection of organisms by culture, histopathology or PCR, even though a negative test for Lyme antibody in CSF cannot rule out the diagnosis [10].

Although only a few cases of optic neuritis caused by Borreliosis have been described, Kubová et al. showed that, in patients with confirmed neuroborreliosis, 42% reported blurred vision or diplopia and 27% had delayed visual evoked potentials, meaning that optic nerve involvement in Lyme disease is probably more common than previously thought [11]. The PCR for *B. burgdorferi* on the CSF was negative in this case, but this test has low sensitivity. Pleocytosis and intrathecal production of IgG are common findings in CNS involvement and the improvement after treatment also supports this diagnosis. We also exhaustively excluded other possible etiologies.

Neuroborreliosis is effectively treated with intravenous antibiotics, including ceftriaxone, doxycycline and penicillin G, for two to four weeks. Reports of Lyme optic neuritis mainly used ceftriaxone. Early recognition and treatment can prevent the persistence of neurological deficits, namely permanent vision loss. However, recovery is slow and may be incomplete in patients with late disease [8].

Follow-up appointments should aim to confirm treatment response and check for post-treatment Lyme disease syndrome (PTLDS), which is described as total or partial recovery from the manifestations of Lyme disease after treatment followed by persistent or relapsing nonspecific symptoms, usually starting more than six months after completion of the antibiotic therapy [6]. Those symptoms are fatigue, myalgias, sleep disturbances, headache, and arthralgia. There is no specific treatment for this syndrome.

## Conclusions

Lyme disease should always be considered in the differential diagnosis of patients from endemic areas with nonspecific symptoms. The diagnosis of neuroborreliosis is challenging but prompt identification and treatment can prevent the development of complications and sequelae.

## References

[1] Aberer E (2007). Lyme borreliosis - an update. J German Soc Dermatol.

[2] Rizzoli A, Hauffe HC, Carpi G, Vourc’h GI, Neteler M, Rosà R (2011). Lyme borreliosis in Europe. Eurosurveillance.

[3] Hildenbrand P, Craven DE, Jones R, Nemeskal P (2009). Lyme neuroborreliosis: manifestations of a rapidly emerging zoonosis. Am J Neuroradiol.

[4] Rahn DW, Felz MW (1998). Lyme disease update: current approach to early disseminated, and late disease. Postgrad Med.

[5] Halperin J (2015). Nervous system lyme disease. Clin Lab Med.

[6] Burakgazi AZ, Henderson CS (2016). Unusual presentation of unilateral isolated probable lyme optic neuritis. Case Rep Neurol Med.

[7] Sibony P, Halperin J, Coyle PK, Patel K (2005). Reactive lyme serology in optic neuritis. J Neuroophthalmol.

[8] Marques R (2015). Lyme neuroborreliosis. Continuum.

[9] Centers for Disease Control and Prevention (1995). Recommendations for test performance and interpretation from the Second National Conference on Serologic Diagnosis of Lyme Disease. Morbid Mortal Wkly Rep.

[10] Jha P, Rodrigues Pereira SG, Thakur A, Jhaj G, Bhandari S (2018). A case of optic neuritis secondary to lyme disease. WMJ.

[11] Kubová Z, Szanyi J, Langrová J, Kremláček J, Kuba M, Honegr K (2006). Motion-onset and pattern-reversal visual evoked potentials in diagnostics of neuroborreliosis. J Clin Neurophysiol.

